# Maternal Reports of Preterm and Sick Term Infants’ Settling, Sleeping and Feeding in the 9 Months after Discharge from Neonatal Nursery: An Observational Study

**DOI:** 10.3390/children11060655

**Published:** 2024-05-28

**Authors:** Emma Shu Min Lim, Julie Williams, Philip Vlaskovsky, Demelza J. Ireland, Donna T. Geddes, Sharon L. Perrella

**Affiliations:** 1 School of Biomedical Sciences, The University of Western Australia, Perth, WA 6009, Australia; 2 Neonatology Clinical Care Unit, King Edward Memorial Hospital, Subiaco, WA 6008, Australia; 3 Department of Mathematics and Statistics, School of Physics, Mathematics and Computing, The University of Western Australia, Perth, WA 6009, Australia; 4 School of Molecular Sciences, The University of Western Australia, Perth, WA 6009, Australia; 5 ABREAST Network, Perth, WA 6000, Australia; 6 UWA Centre for Human Lactation Research and Translation, Perth, WA 6009, Australia

**Keywords:** sleep, sleep duration, infant behaviour, mother, intensive care, neonatal, breastfeeding

## Abstract

The effects of preterm birth, neonatal morbidities and environmental influences on infant sleep development is an important yet under-researched topic, with little known about normative sleep for infants born sick or preterm. The aim of this prospective, observational longitudinal study was to evaluate maternal perceptions and degree of bother with infant sleep behaviours and feeding outcomes across the first 9 months after discharge for sick/preterm infants cared for in the neonatal intensive care unit (NICU) and for healthy term-born infants. This paper reports outcomes for the sick/preterm cohort (*I* = 94) that were recruited from two NICUs in Perth, Western Australia. Total bother scores were on average 20.2% higher at 9 months than at two weeks post-discharge (*p* < 0.001). Increased night waking frequency, evening settling duration and crying duration were all positively associated with total bother scores. Maternal confidence scores were negatively associated with maternal bother scores; with each unit increase in confidence, maternal bother decreased by 8.5% (*p* < 0.001). Covariates such as birth gestation, breastfeeding status and multiple births were not associated with maternal bother. Families may benefit from additional support when experiencing increased night waking frequency and crying and settling durations in the first 9 months after discharge from NICU.

## 1. Introduction

In Western culture, parental perceptions of problematic infant sleep are prevalent [1]. Expectations of infant behaviours such as falling asleep independently and longer sleep durations are at odds with infant biology, and so culturally aligned maternal expectations of “normal” infant sleep can compromise maternal confidence and family wellbeing [2,3]. Mothers of infants born sick and/or preterm are particularly vulnerable to negative perceptions of their infant’s feeding and sleep, which are associated with poorer maternal physical and mental health [4]. Infants who require neonatal intensive care unit (NICU) admission face unique environmental and biological challenges that can impact the establishment of sleep and breastfeeding. Current understandings of “normal” infant sleep do not consider infant health characteristics and may thus be inapplicable to infants born sick or preterm [2]. Knowledge of preterm/sick infant sleep development is needed to inform family guidance and to optimise both maternal and infant wellbeing.

Sleep ontogenesis is a complex process that parallels and interacts with other domains of child development including cognitive and physical maturation [5,6]. Rapid developmental changes to infant sleep occur over the first year of life [1,7,8,9] and are positively associated with learning, memory, executive function, behavioural issues and social competence [2,6]. While essential to lifelong neurological wellbeing [10,11,12,13], there is no universal definition of optimal or “normal” infant sleep [2,7]. Extensive inter-individual variation in sleep and settling patterns are driven by a complex interplay between environmental, genetic and social factors that are not well understood [1,8,14], making “one-size-fits-all” recommendations impractical when considering the unique needs of individual infants [3].

Broadly, “normal” infant sleep may be conceptualised from evolutionary, epidemiological or historico-cultural perspectives [3]. Evolutionary or biological norms anticipate that infants wake regularly during the night for feeding and interaction with their mother; while Western cultural expectations promote sleeping through the night, elimination of night feeds and “establishing a routine” [3]. In cultural contexts, “good” infant sleep is often characterised by “independent sleep onset, longer consolidated sleep periods, self-soothing at night and more sleep per sleep–wake cycle” [2]. When maternal expectations contrast with the realities of normal infant sleep, maternal worry and concern may result [3]. Both biologically and culturally, infant feeding and sleep are closely intertwined [15,16]. Infant physiology necessitates frequent waking to feed throughout the night, fulfilling the nutritional and energetic requirements for rapid infant growth and development [3,15]. Breastfeeding and the circadian rhythmicity of human milk melatonin support infants’ return to sleep [17], while frequent breast milk removal in turn ensures the maintenance of maternal milk production [15]. Common throughout Western culture, however, is the idea that commercial formula milk feeding promotes infant (and thereby maternal) sleep, while breastfeeding is disruptive to sleep [16]. Mothers are challenged with balancing maternal and infant needs, while untangling competing biological and cultural expectations on infant feeding and sleep [16]. Thus, regardless of their infant’s sleep patterns, mothers’ feeding experiences may influence their expectations surrounding infant sleep.

Ideas of problematic infant sleep influence maternal perceptions, yet published definitions of “sleep problems” are as varied and subjective as those for “normal” infant sleep [7]. Published definitions range from deviations from statistical averages for sleep duration to long latency to sleep, short duration of nighttime sleep and frequent night waking [18] to “anything that disturbs the parents or does not match parents’ expectations for sleep” [7]. Night waking, in particular, is commonly cited by parents as a major indicator of “poor” infant sleep [5,16,19], despite fragmented sleep being biologically normal, occurring in ~20–30% of infants during the first 24 months of life [20].

While parent-reported sleep problems are highly prevalent, not all cases are clinically significant, and the over-reporting of infant sleep problems is common [1]. Cultural expectations, maternal mental health, maternal sleep quality, knowledge of infant sleep, confidence in parenting abilities and feeding practices all influence maternal perceptions of infant sleep [19,20]. Therefore, consideration of several maternal and infant characteristics is important when providing individualised infant sleep advice [20].

Sick and preterm newborns admitted to the NICU are exposed to simultaneous sensory overstimulation from light and sound stimuli, medical interventions and infant handling [10,21,22] and under-stimulation from tactile and kinaesthetic deprivation [23]. This atypical environment may disrupt the newborn infant’s complex brain development and sleep ontogenesis [10,13,24]. Furthermore, sleep may be further complicated by co-morbidities such as disordered breathing [11].

The sleep outcomes of infants born sick/preterm are unclear, with great variability in reported study results [22]. Preterm infants may have inconsistent sleep patterns across the first year, which then gradually assimilate to that of healthy term-born counterparts [2]. There is some evidence that infants born preterm have an increased prevalence of abnormal sleep patterns, as well as decreased sleep efficiency and total sleep time relative to full-term infants [10,11,25]. Yet, a review of preterm infant sleep studies [13] found that overall, there was no difference in nocturnal sleep duration between preterm and term-born infants. Variability in study designs and measured sleep parameters makes it difficult to effectively compare the sleep outcomes of term and preterm infants.

Few studies have described the sleep patterns of sick term-born infants admitted to the NICU, and findings of preterm infant studies may not be applicable to this diverse population [26]. Analysis of NICU infant sleep should consider the heterogeneity in developmental maturities and the nature of medical/surgical morbidities between preterm and term sick populations, as well as parents’ perceptions of infant sleep.

The transition to motherhood involves major lifestyle adjustments and is further impacted by the birth of a preterm or sick infant [27]. Maternal expectations of infant sleep, where unrealistic or unmet, can compromise maternal mental health and wellbeing [16]. Parenting a preterm infant is a significantly different experience characterised by early separation and altered parental roles during the infant’s NICU stay [28]. Admission to the NICU is a traumatic stressor for parents [26] and can result in poor sleep, stress, anxiety, depression and fatigue [27,28]. Parents of preterm infants are more likely to report concern with perceived infant sleep problems than parents of term-born infants [13]. Maternal mental health and the ability to meet their sick/preterm infant’s needs are crucial factors in the infant’s long-term development [27], so the provision of adequate guidance and support is important.

Knowledge of the effects of preterm birth, neonatal morbidities and environmental influences on subsequent sleep development is limited [6,13]. Current sleep models and recommendations for term-born infants may not apply to sick/preterm infants [2], yet descriptions of normative sick/preterm infant sleep are lacking. Published preterm infant sleep studies have used various instruments and sleep measures [6,9], with the heterogeneity of their methods limiting their comparability [10]. Most studies were cross-sectional, short-term longitudinal or only conducted during the hospital stay [25], and so an understanding of longitudinal infant sleep development is lacking [27]. Lastly, the sleep development of sick term-born infants who require NICU care is a significantly understudied area.

The aims of this study are to examine a cohort of infants born sick/preterm and admitted to the NICU:

- Maternal perceptions of crying, settling and sleep patterns across the first 9 months after discharge.
- The degree of maternal bother with infant sleep, settling and crying duration and night waking frequency across the first 9 months after discharge.
- Maternal breastfeeding self-efficacy.

## 2. Materials and Methods

### 2.1. Research Design

A prospective observational longitudinal study was conducted to investigate the feeding and sleeping patterns in cohorts of sick/preterm infants (admitted to the NICU) and healthy term infants, across the first 9 months after hospital discharge. The primary aim of this study was to describe and compare breastfeeding outcomes between the two cohorts, previously reported by Perrella et al. [29] A secondary aim was to describe infant sleep and settling behaviours, the degree of maternal bother associated with these behaviours and maternal breastfeeding confidence and satisfaction of the sick/preterm and healthy cohorts across the first 9 months following hospital discharge. Outcomes of the healthy term cohort have previously been reported [30]. The focus of this paper will be to describe the outcomes of the sick/preterm cohort.

### 2.2. Sample Size

An estimated sample of 70 mothers in each of the sick/preterm and healthy term cohorts was calculated for the study’s primary aim of detecting a 25% difference in breastfeeding rates with 86% power [29]. To account for an attrition rate of approximately 30%, the total sample size was increased to 180 [29].

### 2.3. Participants and Recruitment

Participants were recruited between October 2006 and July 2007 from King Edward Memorial Hospital (KEMH) and Princess Margaret Hospital (PMH) in Perth, Western Australia. Using a convenience sample of all mothers who met the study criteria, a cohort of 91 mothers of healthy term newborn infants was recruited from the KEMH postnatal wards, while 94 mothers of sick/preterm infants were recruited from the NICUs at KEMH and PMH following ≥5 days of admission. Inclusion criteria were English-speaking mothers ≥18 years of age, who were breastfeeding at the time of discharge and intended to continue. Participants also had to be accessible via telephone for follow-up interviews up to 9 months after discharge. Exclusion criteria were mothers who were non-English speaking, <18 years of age, not intending to breastfeed and/or not accessible via telephone for up to 9 months after discharge. Prospective participants were provided with verbal and written study information, and informed written consent was obtained. This study was approved by the Ethics Committee of the Women’s and Children’s Health Service (reference EC06-05).

### 2.4. Data Collection

Upon recruitment at 24 to 48 h prior to discharge, baseline demographic, infant health and feeding data were collected. Maternal intended breastfeeding duration and previous breastfeeding duration were collected, and the Breastfeeding Self-Efficacy Scale-Short Form (BSES-SF) was completed by participants.

**Figure 1:**
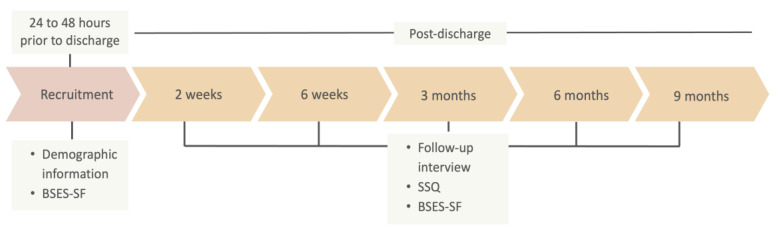
Study flow diagram.

Follow-up was conducted via scheduled telephone interviews at 2 weeks, 6 weeks, 3 months, 6 months and 9 months following discharge by a research assistant with appropriate knowledge and skills. Infant feeding practices were recorded and the Sleep and Settle Questionnaire (SSQ) was administered at each time point, with mothers asked to report outcomes “over the last week”. The BSES-SF was administered if the mother was still breastfeeding (Figure 1). As the aim of the study was to assess maternal perceptions of infant behaviours, no attempt was made to address the bias inherent in subjective reports of behaviour.

### 2.5. Instruments

The following instruments and outcome measures were administered.

#### 2.5.1. Demographic and Infant Health Information

A demographic, infant health and feeding questionnaire was administered 24 to 48 h prior to discharge. Recorded infant characteristics included gestational age, reason for admission to the NICU and length of hospital admission. Data on maternal characteristics and breastfeeding history were also collected. Socio-economic status was classified using Socio-Economic Indexes for Areas (SEIFA), which ranks relative socio-economic disadvantage by postal code [31].

#### 2.5.2. Breastfeeding Self-Efficacy Scale-Short Form (BSES-SF)

The BSES-SF assesses maternal confidence in the ability to breastfeed, with early postpartum BSES-SF scores predictive of breastfeeding initiation and duration [32]. It contains 14 positively worded items, including “I can always determine that my baby is getting enough milk” and “I can always be satisfied with my breastfeeding experience”. Items are scored using a five-point Likert scale (1 = “not at all confident” to 5 = “always confident”). Responses are summed to produce a range from 14 to 70, where higher scores indicate higher levels of breastfeeding self-efficacy. The BSES-SF has reported high reliability, with a Cronbach’s alpha coefficient of 0.94 [32].

#### 2.5.3. Follow-Up Interview

An infant feeding questionnaire was administered with items including feeding method (fully breastfed (BF), mixed; both BF and formula, or formula only), the timing of introduction of solid foods and the date of breastfeeding cessation.

#### 2.5.4. Sleep and Settle Questionnaire (SSQ)

The SSQ is designed to assess parental perceptions of infant sleep and settling behaviours and the degree of “bother” or concern associated with these behaviours [33]. It is the only validated instrument that assesses parents’ emotional responses to perceptions of infant sleep [33]. It assesses a parent’s perceptions of 34 infant behaviours over the past week, including durations of crying, settling and sleep. Some items are specific to the daytime (0500 to 1800 h), evening (1800 to 2200 h) or nighttime (2200 to 0500 h). Nine items assess the degree of bother the parents experience in relation to infant behaviours, using a five-point Likert scale (1 = “didn’t bother me at all” to 5 = “bothered me extremely”). Responses are summed to produce an overall bother score (range 9 to 45, >27 = “bothered”) with higher scores indicating a higher degree of bother. Two items assess maternal confidence in their ability to calm and settle their baby (score range 2 to 10), while one item assesses maternal confidence in their partner’s ability to do the same (score range 1 to 5). The SSQ has demonstrated moderate test–retest reliability and good validity [33].

## 3. Data Analysis

Data were summarized with means and standard deviations, medians and lower and upper quartiles or counts and proportions as appropriate to summarise demographic characteristics, infant sleep and settling patterns, maternal bother scores, confidence scores, feeding outcomes and BSES-SF scores. Participants were classified into three groups based on birth gestation: preterm < 33 weeks, preterm 33 to 36.9 weeks and term sick ≥ 37 weeks. Outcomes described for the entire cohort and by birth gestation group.

Linear mixed modelling was used to examine a range of factors influencing the outcome of maternal bother over time, such as infant crying, settling and sleep, feeding and maternal characteristics. As per the analysis of the healthy term cohort [30], a linear mixed model was fitted with total bother score as the response and individual infant as a random effect. Univariable associations were assessed, and purposeful selection with a *p*-value cut-off of 0.1 was used to arrive at a subset of covariates for a multivariable model. Backward elimination was subsequently used to arrive at a final multivariable model for maternal bother. Of particular interest was the examination by birth gestation group with adjustment for other covariates, some of which were considered to be potential confounders. This included sleep, settling and crying durations, night-waking frequency, feeding method, multiple births, maternal characteristics (age, education, marital status, and ethnicity), parity, intended breastfeeding duration, total confidence score and confidence in partner score. BSES-SF score was found to be an unviable covariate due to significant sample loss beyond the 6-week follow-up timepoint.

The outcome was a natural log transformed to satisfy regression assumptions related to the constant variance of the residuals, resulting in coefficients that are multiplicative in nature. Sleep and settling durations were log base 2 transformed due to their heavily skewed distributions, with 1 added prior to logging to address 0 values. Final statistical analysis was performed by a statistician using R (R version 4.3.1, http://www.r-project.org, accessed on 5 June 2023).) with statistical significance set at *p* < 0.05.

## 4. Results

Maternal demographic and background feeding characteristics are presented in Table 1, and infant demographic characteristics are presented in Table 2. Of the 103 mothers recruited, 9 were excluded due to missing demographic information or loss of follow-up at all 5 timepoints. This resulted in a final sample of 94 mothers of sick/preterm infants.

The study population included 101 sick/preterm infants, of which 82 were singleton infants, and 19 were twin infants (7 twin pairs and 5 individual twin infants). Participants were classified into three groups based on birth gestational age: preterm < 33 weeks, preterm 33 to 36.9 weeks and term sick (≥40 weeks).

Maternal BSES-SF scores and the feeding method across the first 9 months are reported in Table 3. On average, breastfeeding confidence increased over time. However, some breastfeeding mothers did not complete all BSES-SF items, particularly item 11 “I can always finish feeding my baby on one breast before switching to the other breast” as this was not applicable to mothers of breastfeeding twins, to mothers of infants who were satisfied with feeding from only one breast. Incomplete BSES-SF responses were excluded from the analysis, resulting in significant sample loss over time. While all mothers were providing breast milk at the time of discharge, breastfeeding cessation was reported by a quarter of mothers at 6 weeks and half by 6 months after NICU discharge (Table 3).

Maternal reports of infant crying, settling and sleep behaviours across the first 9 months following hospital discharge are summarised in Table 4, with characteristics reported by birth gestation group in Table A1. Over time, daytime sleep frequency and duration of morning and afternoon sleep decreased, while the duration of evening and night sleep increased. Night waking was reported at all time points and decreased over time. Settling durations in the day, evening and night all showed great inter-individual variability, with a downward trend over time. Crying durations were similarly variable, though mothers reported the longest daytime and evening crying durations at 6 weeks. Mothers typically reported low degrees of bother and high levels of confidence in both their own and their partner’s abilities to settle their infant across the first 9 months.

**Figure 2:**
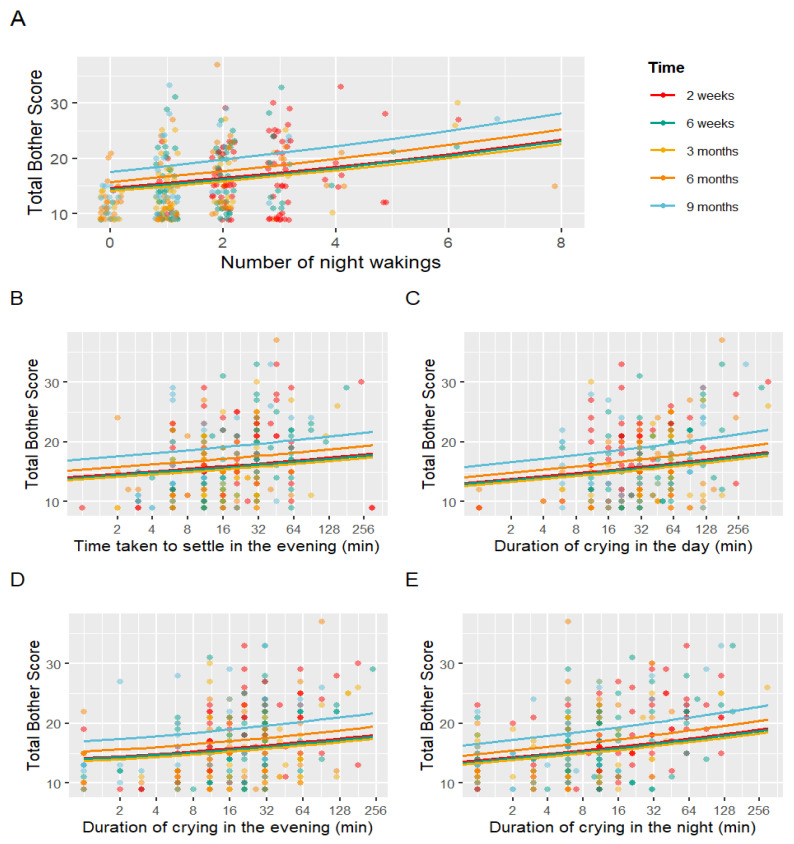
Linear mixed models demonstrating associations with maternal bother. Figures demonstrate relationships between total bother scores and night-waking frequency (**A**), time taken to settle in the evening (**B**), duration of crying in the day (**C**), duration of crying in the evening (**D**), and duration of crying in the night (**E**).

A multivariable model examining the influence of sleep variables on maternal bother scores over time was produced, with the intercept as total bother scores at 2 weeks. Outcomes significantly associated with maternal bother are reported in Table 5. Coefficients are multiplicative in nature. At 9 months, total bother scores were on average 20.2% higher than at 2 weeks (*p* < 0.001). Increased night-waking frequency (Figure 2A), increased settling duration in the evening (Figure 2B) and increased crying duration (Figure 2C–E) were associated with increased total bother scores. With each night waking, maternal bother increased by 6.0% (*p* < 0.001); and with every doubling of nighttime crying duration, maternal bother increased by 4.3% (*p* < 0.001).

Total confidence scores and total confidence in partner scores were associated with decreased total bother scores. Mothers with higher confidence scores reported lower maternal bother, and with each unit increase in confidence, maternal bother decreased by 8.5% (*p* < 0.001). All other covariates examined, such as birth gestation group (*p* = 0.439), exclusive breastfeeding (*p* = 0.436) and multiple births (*p* = 0.763), were not associated with maternal bother.

## 5. Discussion

Our evaluation of the relationships between maternal bother and maternal perceptions of sick/preterm infant feeding, crying, settling and sleep found associations with night-waking frequency and durations of crying and evening settling (Table 5). Breastfeeding was not associated with maternal bother, although half of the mothers had weaned by 6 months post-discharge. While current reports of sick/preterm infant sleep patterns are conflicting, we found that mothers typically had high levels of confidence and low levels of bother in regard to their preterm and term sick infants’ behaviours across the 9 months after discharge from NICU.

### 5.1. Biologically “Normal”

Maternal reports suggested sick/preterm infant crying, settling and sleep patterns aligned with biological norms previously reported in healthy term infants. Mothers in our study reported a shift towards nocturnal sleep over the first 9 months, with decreasing daytime sleep durations and increasing nighttime sleep durations paralleling the development of their infants’ circadian rhythm [6]. Night waking at all time points and variability in crying and settling durations were within expectations of “normal” infant sleep [3,6]. Daytime and evening crying durations peaked at 6 weeks, consistent with published reports [34]. Overall, our cohort reported low maternal bother and high confidence in both their own and their partner’s ability to settle their infant (Table 4).

Using the same SSQ measures in a cohort of mothers of healthy term infants, Perrella et al. [30] observed similar trends with increasing nighttime sleep over time, night waking at all timepoints and highly variable crying and settling durations [30]. While these findings are consistent with previous studies that found no difference between preterm and healthy full-term infant sleep, the majority of these studies reported on infant sleep patterns > 9 months post-hospital discharge [13,35].

### 5.2. Maternal Bother with Sick/Preterm Infant Sleep

Our evaluation of the relationship between maternal bother and sick/preterm infant sleep found significant associations with night-waking frequency and durations of crying and evening settling (Table 5). Crying at any time was associated with increased maternal bother scores, but especially at night. Night waking and infant crying are key factors associated with parental perceptions of problematic infant sleep [5,16,19] and have even demonstrated links with maternal depression and anxiety [36,37,38]. Although common and biologically normal, infant crying and night waking require more active nocturnal care and can disrupt maternal sleep [38,39], while increased settling duration and infants’ increased need for attention can exacerbate maternal stress [33,40]. It is thus unsurprising that they caused notable increases in maternal bother within our cohort.

Maternal bother was significantly higher at 9 months than at 2 weeks post-discharge (Table 5). It is possible that in the weeks after discharge, mothers may have had lower expectations and greater tolerance of their infant’s behaviours, especially since NICU graduates have previously been described as fussier and less adaptable to changes [41]. However, this tolerance may diminish over time, especially if fussy infant behaviours persist over many months.

Higher maternal confidence in their own and their partner’s ability to settle their infant was associated with decreased maternal bother scores within our cohort (Table 5). It has been reported that confidence regulates thought-induced stress, and mothers with lower confidence in their parenting abilities may perceive a wider range of infant behaviours as problematic [30,42]. Thus, mothers with higher confidence may have more positive perceptions of their infant’s sleep behaviours and report less bother.

It is clear maternal expectations and perceptions mediate their emotional responses to infant sleep [3]. Mothers of NICU graduates may be more vulnerable to stress, and so anticipatory guidance regarding sick/preterm infant behaviours is important.

#### 5.2.1. Maternal Bother in Sick/Preterm vs. Healthy Term Cohorts

Mothers of healthy term infants reported similar degrees of bother in relation to infant sleep variables as those of preterm and sick term infants. Perrella et al. [30] observed mothers of healthy term infants were similarly most bothered by increased night-waking frequency and durations of crying. While both cohorts were bothered by durations of settling, daytime settling rather than evening settling was significantly associated with the healthy term cohort [30]. Despite variability in infant characteristics, mothers were similarly bothered by infant behaviours that required their attention throughout the day (but especially at night) and which likely compromised their own sleep or ability to complete other tasks. Our findings suggest mothers of sick/preterm and healthy term infants had similar perceptions of infant crying, settling and sleep patterns. The similarity in bother scores between the two cohorts (<17 in sick/preterm, <18 in healthy term) contrasts with previous reports of parents of preterm infants being more concerned about infant sleep [13,35]. Mothers of NICU infants typically experience a period of prolonged anxiety over their infant’s health and survival [28,41] and may respond to their “fragile and vulnerable” infant with greater concern and in an “oversolicitous” manner [22]. Thus, it is possible that mothers’ concern over their sick/preterm infant’s health may take precedence over sleep concerns, thus mediating maternal bother.

#### 5.2.2. Maternal Bother in Preterm vs. Term Sick Infants

Interestingly, the birth gestation group was not found to be associated with maternal bother (Table 5), suggesting no detectable difference in maternal perceptions of sleep between preterm infants born at <33 weeks and 33–36.9 weeks gestation and term sick infants (Appendix A). While there is a wide body of literature on the perceptions and experiences of parents of preterm infants, the great heterogeneity in infant characteristics between preterm and term sick infants in the NICU has seen researchers wary of generalising findings between the groups [26]. Yet, reports of sick infants born at term are scarce in the current infant sleep literature [26]. Our findings suggest that mothers of preterm and term sick infants have similar needs, stressors and experiences, which contribute to similar perceptions of infant sleep and subsequent bother. However, further investigation with a larger sample size is needed to confidently assess the impact of variability in infant characteristics on maternal perceptions of infant sleep and subsequent bother.

### 5.3. Breastfeeding Confidence in Mothers of Sick/Preterm Infants

Despite providing breast milk at discharge, one-third of mothers were exclusively breastfeeding at 6 months, with half having weaned to exclusive formula feeding (Table 3). Mean BSES-SF scores were 57.9 at discharge and higher than the threshold of <58 for low confidence beyond discharge home (Table 3). As low early BSES-SF is associated with a shorter breastfeeding duration [43], it is unsurprising that approximately 50% of our cohort had weaned by 6 months, with many reporting low BSES-SF scores at discharge.

Although studies have suggested that the challenge of breastfeeding a NICU graduate infant can cause maternal stress and anxiety [44,45,46], feeding method was not found to be associated with maternal bother in this cohort (Table 5). This was also described in the term healthy cohort [30], indicating that breastfeeding mothers were not more bothered by their infant’s sleep than formula-feeding mothers. While contrasts with a study where cultural perspectives on the relationship between infant feeding and sleep were reported to influence maternal infant feeding decisions [16], our findings suggest cultural differences between British and Australian mothers’ attitudes towards breastfeeding.

### 5.4. Limitations

“Bother” is not a direct measure of maternal mental health, and although maternal mental health closely impacts perceptions of infant sleep [39], our study did not assess perinatal mental health status. Furthermore, bother was only assessed at distinct timepoints and did not account for periods of transition of feeding method. The BSES-SF was not completed by all mothers of our cohort, particularly mothers of twins, limiting the ability to assess breastfeeding self-efficacy in relation to maternal bother. Finally, our cohort primarily consisted of partnered, Caucasian, educated mothers, which limits the generalisability of these findings to diverse populations. In focusing on maternal outcomes, our study did not account for the unique experiences of fathers in parenting sick/preterm infants.

## 6. Conclusions

Infant feeding, crying, settling and sleep are of major concern to parents of infants born sick or preterm. Increased night waking and crying and settling durations had positive associations with maternal bother across the first 9 months. Our study adds to the limited evidence on maternal perceptions of infant feeding and sleep in sick/preterm infants, with the opportunity for comparison between full-term, preterm and term sick infant populations. Our findings indicate that mothers of infants born sick or preterm have similar perceptions of infant feeding and sleep to those of healthy term-born infants. However, further studies are needed to inform individualised anticipatory guidance and support in this vulnerable population of mothers and infants post NICU discharge.

## Figures and Tables

**Table 1:** Maternal demographic and background feeding characteristics.

Maternal Characteristics *n* (%)	<33 Weeks (*n* = 35)	33–36.9 Weeks (*n* = 22)	Term Sick (*n* = 37)	Overall (*N* = 94)
Age				
<30 years	19 (54.3)	9 (40.9)	14 (37.8)	42 (44.7)
≥30 years	16 (45.8)	12 (54.5)	23 (62.2)	51 (54.3)
Missing	0 (0)	1 (4.5)	0 (0)	1 (1.1)
Highest education level				
High school	11 (31.4)	7 (31.8)	18 (48.6)	36 (38.3)
TAFE ^1^/diploma	15 (42.9)	7 (31.8)	6 (16.2)	28 (29.8)
Tertiary	9 (25.7)	8 (36.4)	13 (35.1)	30 (31.9)
Marital status				
Married/de facto	34 (97.1)	21 (95.5)	36 (97.3)	91 (96.8)
Ethnic group				
Aboriginal/Torres Strait Islander	1 (2.9)	2 (9.1)	2 (5.4)	5 (5.3)
Caucasian	28 (80.0)	20 (90.9)	31 (83.8)	79 (84.0)
Asian	2 (5.7)	0 (0)	3 (8.1)	5 (5.3)
Other	3 (8.6)	0 (0)	0 (0)	3 (3.2)
Missing	1 (2.9)	0 (0)	1 (2.7)	2 (2.1)
Breastfed previously	12 (34.3)	12 (54.5)	19 (51.4)	43 (45.7)
Missing	0 (0)	0 (0)	1 (2.7)	1 (1.1)
Intended breastfeeding duration				
0–6 months	4 (11.4)	8 (36.4)	6 (16.2)	18 (19.1)
6–12 months	16 (45.7)	8 (36.4)	25 (67.6)	49 (52.1)
>12 months	6 (17.2)	3 (13.6)	4 (10.8)	13 (13.9)
“As long as I can/I want”	8 (22.9)	2 (9.1)	0 (0)	10 (10.6)
Missing	1 (2.9)	1 (4.5)	2 (5.4)	4 (4.3)

^1^ TAFE, technical and further (non-tertiary) education.

**Table 2:** Infant demographic characteristics.

Infant Characteristics *n* (%)	<33 Weeks (*n* = 37)	33–36.9 Weeks (*n* = 26)	Term Sick (*n* = 38)	Overall (*N* = 101)
Infant sex = female	19 (51.4)	14 (53.8)	20 (60.5)	56 (55.4)
Twin infants	5 (13.5)	10 (38.5)	4 (10.5)	19 (18.8)
Birth gestation (weeks)				
Median (Q1, Q3)	28.4 (27.0, 30.9)	35.0 (33.9, 35.0)	39.3 (38.1, 40.0)	35.0 (30.4, 68.6)
Age at discharge (days)				
Median (Q1, Q3)	62.0 (37.0, 83.0)	13.5 (9.75, 17.3)	13.0 (8.0, 21.0)	22.5 (11.0, 61.5)
Missing *n* (%)	0 (0)	2 (7.7)	9 (23.7)	11 (10.9)
Length of NICU admission (days)				
Median (Q1, Q3)	64.0 (37.0, 83.0)	15.0 (9.0, 18.0)	9.0 (5.0, 13.8)	21.0 (9.0, 62.5)
Missing *n* (%)	0 (0)	5 (19.2)	8 (21.1)	13 (12.9)

**Table 3:** Breastfeeding self-efficacy (BSES-SF) scores and feeding outcomes across the first 9 months after NICU discharge.

Feeding Outcomes *N* = 101	Discharge	2 Weeks	6 Weeks	3 Months	6 Months	9 Months
BSES-SF score	*n* = 92	*n* = 68	*n* = 62	*n* = 48	*n* = 39	*n* = 24
M ± SD	57.9 ± 9.3	59.1 ± 8.9	60.9 ± 8.1	62.9 ± 8.2	64.5 ± 6.4	64.4 ± 6.1
Feeding method *n* (%)		*n* = 96	*n* = 98	*n* = 99	*n* = 98	*n* = 94
Fully breastfeeding		66 (65.3)	46 (45.5)	35 (34.7)	35 (34.7)	24 (23.8)
Mixed feeding		21 (20.8)	25 (24.8)	26 (25.7)	13 (12.9)	9 (8.9)
Formula feeding		9 (8.9)	27 (26.7)	38 (37.6)	50 (49.5)	61 (60.4)

**Table 4:** Sleep and Settle Questionnaire (SSQ) outcomes across the first 9 months.

SSQ Items (M ± SD) *N* = 101	2 Weeks (*n* = 97)	6 Weeks (*n* = 96)	3 Months (*n* = 93)	6 Months (*n* = 93)	9 Months *(n* = 85)
Sleep duration (h)					
Morning sleep	2.5 ± 1.0	2.4 ± 1.0	2.2 ± 1.2	1.4 ± 0.8	1.4 ± 0.6
Afternoon sleep	2.4 ± 1.1	2.3 ± 1.0	1.9 ± 1.0	1.5 ± 0.8	1.4 ± 0.7
Evening sleep	2.2 ± 1.0	2.2 ± 0.9	1.9 ± 1.1	2.2 ± 1.0	2.5 ± 0.9
Night sleep	3.1 ± 1.2	4.2 ± 1.5	5.6 ± 1.6	5.9 ± 1.5	6.6 ± 1.0
Daytime sleep frequency	3.8 ± 1.0	3.3 ± 1.0	3.0 ± 0.8	2.5 ± 0.8	2.0 ± 0.4
Night-waking frequency	2.6 ± 1.2	1.8 ± 1.0	1.2 ± 1.2	1.2 ± 1.4	0.8 ± 1.1
Settling duration (min)					
Daytime	24.2 ± 44.0	23.5 ± 27.0	15.8 ± 15.2	12.8 ± 11.8	10.8 ± 10.5
Evening	30.0 ± 48.8	27.6 ± 29.5	25.6 ± 32.0	11.9 ± 11.3	13.0 ± 14.9
Night	29.0 ± 47.4	20.0 ± 24.2	16.3 ± 24.3	8.1 ± 10.2	10.9 ± 16.9
Crying duration (min)					
Daytime	38.7 ± 59.0	55.2 ± 67.4	48.5 ± 58.4	44.4 ± 41.8	41.8 ± 47.8
Evening	24.5 ± 31.3	36.6 ± 53.7	32.2 ± 35.1	19.0 ± 20.6	15.3 ± 19.0
Night	24.4 ± 28.8	24.0 ± 51.4	14.8 ± 39.5	5.7 ± 14.5	7.4 ± 18.5
Total bother score (9–45)	16.6 ± 6.0	16.1 ± 6.6	14.4 ± 5.1	14.3 ± 5.4	15.1 ± 6.1
Total confidence score (2–10)	9.0 ± 1.0	9.2 ± 0.9	9.3 ± 0.9	9.5 ± 0.8	9.4 ± 0.8
Total confidence in partner score (1–5)	3.9 ± 1.0	3.7 ± 1.0	3.8 ± 1.1	3.7 ± 1.1	3.8 ± 1.0

**Table 5:** Infant behaviour variables associated with maternal total bother scores.

Fixed Effects	Estimate	SE	*p*-Value
(Intercept)	20.501	0.182	<0.001
Time (6 weeks)	0.987	0.034	0.701
Time (3 months)	0.965	0.040	0.376
Time (6 months)	1.077	0.041	0.071
Time (9 months)	1.202	0.044	<0.001
Night-waking frequency	1.060	0.013	<0.001
Settling duration (evening)	1.031	0.012	0.010
Crying duration (day)	1.038	0.012	0.001
Crying duration (evening)	1.035	0.010	<0.001
Crying duration (night)	1.043	0.009	<0.001
Total confidence score	0.915	0.018	<0.001
Total confidence in partner score	0.947	0.015	<0.001

## Data Availability

The data that support the findings of this study are available on request from the corresponding author (S.L.P) due to privacy reasons.

## Appendix A

Sleep and Settle Questionnaire (SSQ) outcomes across the first 9 months after NICU discharge reported by birth gestation group.

**Table A1:** Sleep and Settle Questionnaire (SSQ) outcomes across the first 9 months after NICU discharge reported by birth gestation group.

SSQ Items (M ± SD) *N* = 101	2 Weeks (*n* = 97)			6 Weeks (*n* = 96)			3 Months (*n* = 93)			6 Months (*n* = 93)			9 Months (*n* = 85)		
	<33 Weeks (*n* = 37)	33–36.9 Weeks (*n* = 25)	Term Sick (*n* = 35)	<33 Weeks (*n* = 37)	33–36.9 Weeks (*n* = 24)	Term Sick (*n* = 35)	<33 Weeks (*n* = 35)	33–36.9 Weeks (*n* = 22)	Term Sick (*n* = 36)	<33 Weeks (*n* = 35)	33–36.9 Weeks (*n* = 22)	Term Sick (*n* = 36)	<33 Weeks (*n* = 31)	33–36.9 Weeks (*n* = 21)	Term Sick (*n* = 33)
Sleep duration (h)															
Morning sleep	2.7 ± 1.1	2.3 ± 0.8	2.3 ± 1.1	2.7 ± 1.2	2.4 ± 0.9	2.1 ± 0.9	2.5 ± 1.4	2.1 ± 1.2	2.1 ± 1.0	1.5 ± 0.9	1.4 ± 0.9	1.3 ± 0.7	1.4 ± 0.7	1.3 ± 0.6	1.4 ± 0.6
Afternoon sleep	2.6 ± 1.3	2.3 ± 0.8	2.2 ± 0.9	2.5 ± 0.9	2.3 ± 0.8	2.2 ± 1.1	1.9 ± 0.9	2.1 ± 0.7	1.8 ± 1.1	1.5 ± 0.8	1.7 ± 0.7	1.4 ± 0.7	1.5 ± 0.7	1.1 ± 0.6	1.6 ± 0.8
Evening sleep	2.3 ± 0.9	2.2 ± 1.0	2.1 ± 1.1	2.3 ± 0.9	2.1 ± 0.8	2.3 ± 1.0	1.5 ± 1.0	1.7 ± 1.0	2.4 ± 1.0	2.2 ± 1.1	2.1 ± 1.0	2.4 ± 1.0	2.3 ± 1.0	2.7 ± 0.6	2.6 ± 0.9
Night sleep	2.9 ± 1.2	2.8 ± 1.0	3.5 ± 1.1	3.8 ± 1.5	3.7 ± 1.3	5.0 ± 1.2	5.5 ± 1.8	5.4 ± 1.5	5.8 ± 1.4	6.3 ± 1.4	5.5 ± 1.5	5.9 ± 1.5	6.5 ± 1.1	6.5 ± 1.1	6.7 ± 0.9
Daytime sleep frequency	4.0 ± 1.1	3.8 ± 0.9	3.6 ± 1.0	3.3 ± 1.2	3.5 ± 0.8	3.1 ± 0.9	3.1 ± 0.8	3.1 ± 0.7	2.9 ± 0.9	2.5 ± 0.9	2.9 ± 0.8	2.3 ± 0.7	1.9 ± 0.4	2.1 ± 0.3	1.9 ± 0.5
Night-waking frequency	2.8 ± 1.6	2.8 ± 0.7	2.1 ± 0.9	1.9 ± 1.1	2.1 ± 0.9	1.4 ± 0.9	1.3 ± 1.5	1.3 ± 1.4	1.0 ± 0.9	0.6 ± 0.8	1.5 ± 1.6	1.5 ± 1.6	0.5 ± 0.7	1.1 ± 0.7	0.7 ± 1.4
Settling duration (min)															
Daytime	38.1 ± 68.1	13.0 ± 11.1	17.9 ± 14.2	24.4 ± 22.9	17.9 ± 13.3	26.3 ± 35.5	19.0 ± 18.8	14.3 ± 12.6	13.7 ± 12.5	14.8 ± 12.2	11.0 ± 6.4	12.0 ± 13.8	11.7 ± 11.1	9.0 ± 6.4	11.0 ± 12.0
Evening	39.2 ± 66.0	21.6 ± 21.2	26.4 ± 40.9	28.3 ± 24.9	27.0 ± 29.1	27.2 ± 34.9	34.1 ± 40.9	25.5 ± 31.5	18.3 ± 20.5	13.6 ± 12.8	10.2 ± 6.3	11.3 ± 12.3	13.4 ± 13.0	13.2 ± 14.6	12.5 ± 17.1
Night	47.7 ± 70.3	22.4 ± 22.4	14.4 ± 14.3	24.1 ± 25.4	22.8 ± 31.6	13.9 ± 16.4	23.7 ± 27.4	18.1 ± 32.9	8.1 ± 6.2	8.5 ± 9.6	7.7 ± 8.8	8.1 ± 11.5	13.8 ± 17.5	9.5 ± 14.0	9.2 ± 18.9
Crying duration (min)															
Daytime	31.9 ± 39.7	34.5 ± 46.3	49.0 ± 80.6	48.1 ± 43.5	62.7 ± 86.1	57.5 ± 75.0	43.7 ± 34.3	65.7 ± 98.5	42.6 ± 41.1	49.1 ± 41.2	48.4 ± 41.7	37.4 ± 42.5	40.6 ± 36.6	43.6 ± 40.9	41.7 ± 60.8
Evening	18.9 ± 20.9	31.4 ± 38.8	25.5 ± 34.1	33.0 ± 45.2	55.6 ± 77.1	27.3 ± 40.3	32.1 ± 32.1	49.5 ± 49.6	21.3 ± 20.8	23.6 ± 27.0	20.2 ± 17.2	13.9 ± 13.7	14.9 ± 17.1	17.9 ± 17.7	14.1 ± 21.7
Night	25.9 ± 27.8	31.2 ± 37.9	18.1 ± 20.6	22.9 ± 31.4	48.7 ± 91.4	7.4 ± 8.5	10.5 ± 12.6	36.9 ± 75.4	4.6 ± 4.5	7.4 ± 21.7	3.6 ± 7.1	5.3 ± 8.8	3.6 ± 7.9	12.7 ± 23.4	7.6 ± 21.6
Total bother score	17.7 ± 6.3	16.1 ± 5.3	15.9 ± 6.2	16.5 ± 5.9	17.6 ± 8.9	14.7 ± 5.1	14.8 ± 5.6	15.9 ± 5.3	13.1 ± 4.1	13.8 ± 6.1	15.3 ± 4.8	14.3 ± 5.1	14.5 ± 6.5	16.1 ± 5.5	14.9 ± 6.1
Total confidence score	9.1 ± 1.2	9.1 ± 0.9	8.9 ± 0.9	9.2 ± 0.8	9.1 ± 1.1	9.1 ± 0.9	9.4 ± 0.7	9.0 ± 0.8	9.4 ± 0.9	9.6 ± 0.8	9.2 ± 1.0	9.6 ± 0.7	9.5 ± 0.7	9.0 ± 1.0	9.5 ± 0.8
Total confidence in partner score	4.1 ± 1.0	3.8 ± 1.1	3.7 ± 0.9	4.0 ± 1.2	3.5 ± 0.9	3.6 ± 1.0	3.7 ± 1.2	3.8 ± 0.9	3.8 ± 1.1	3.7 ± 1.2	3.7 ± 1.0	3.7 ± 1.1	4.0 ± 1.2	3.9 ± 0.8	3.7 ± 1.0
